# Supplementary material for: The causal role of circulating inflammatory markers in osteoporosis: a bidirectional Mendelian randomized study
Source: Front Immunol. 2024 Jul 18;15:1412298. doi: 10.3389/fimmu.2024.1412298 (PMC11291241; doi:10.3389/fimmu.2024.1412298)
Supplement: Supplementary file 1 [file DataSheet_1.zip › supplementary material/CIM-OSTEOPOROSIS /GCST90274782.csv_forest.pdf]

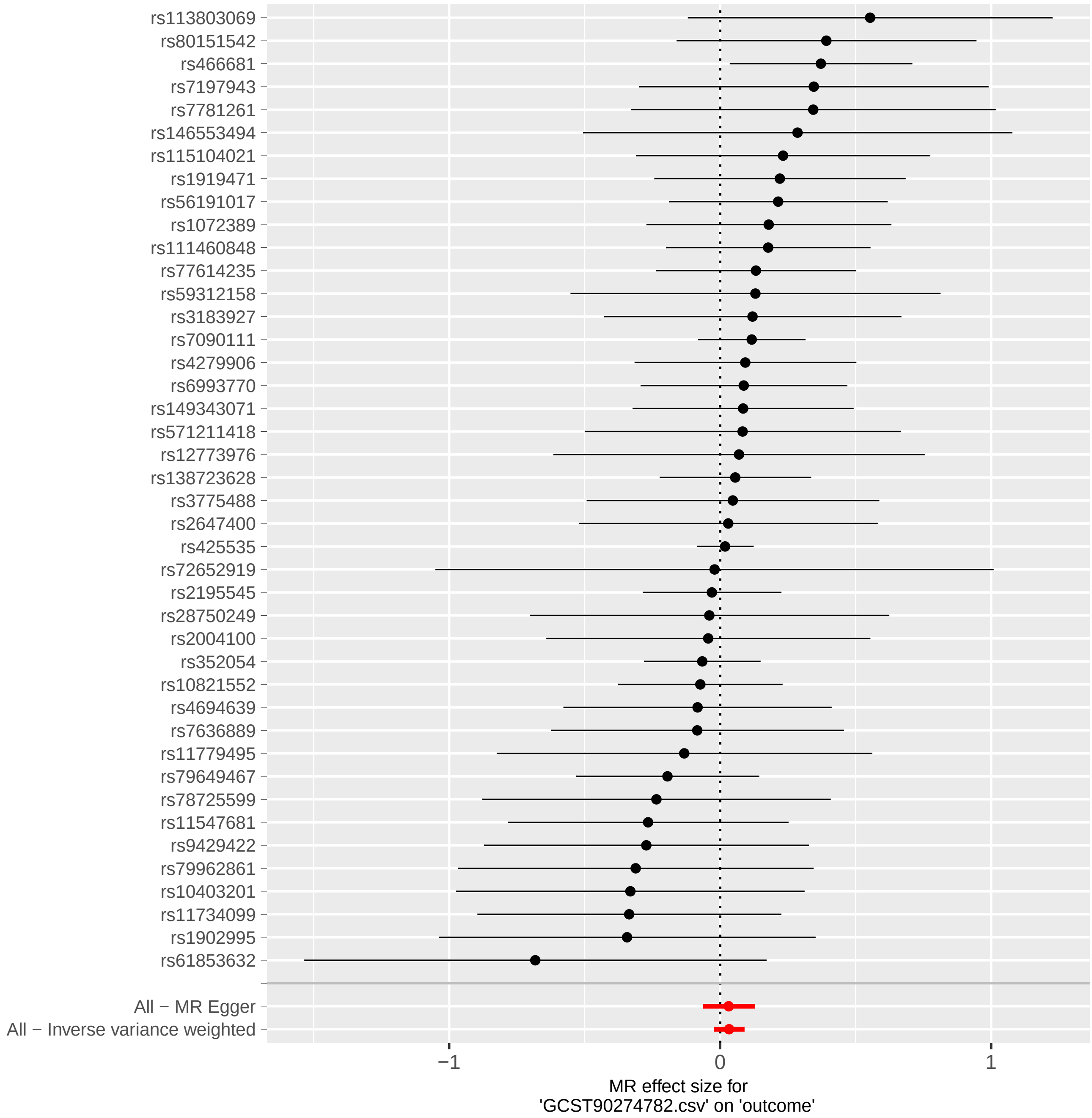

rs113803069
rs80151542
rs466681
rs7197943
rs7781261
rs146553494
rs115104021
rs1919471
rs56191017
rs1072389
rs111460848
rs77614235
rs59312158
rs3183927
rs7090111
rs4279906
rs6993770
rs149343071
rs571211418
rs12773976
rs138723628
rs3775488
rs2647400
rs425535
rs72652919
rs2195545
rs28750249
rs2004100
rs352054
rs10821552
rs4694639
rs7636889
rs11779495
rs79649467
rs78725599
rs11547681
rs9429422
rs79962861
rs10403201
rs11734099
rs1902995
rs61853632
All − MR Egger
All − Inverse variance weighted
−1
0
1
MR effect size for
'GCST90274782.csv' on 'outcome'
